# Sulfur Transport and Metabolism in Legume Root Nodules

**DOI:** 10.3389/fpls.2018.01434

**Published:** 2018-10-10

**Authors:** Manuel Becana, Stefanie Wienkoop, Manuel A. Matamoros

**Affiliations:** ^1^Estación Experimental de Aula Dei, Consejo Superior de Investigaciones Científicas, Zaragoza, Spain; ^2^Department of Ecogenomics and Systems Biology, University of Vienna, Vienna, Austria

**Keywords:** bacteroids, cysteine, (homo)glutathione, legume nodules, sulfur metabolism, symbiosis

## Abstract

Sulfur is an essential nutrient in plants as a constituent element of some amino acids, metal cofactors, coenzymes, and secondary metabolites. Not surprisingly, sulfur deficiency decreases plant growth, photosynthesis, and seed yield in both legumes and non-legumes. In nodulated legumes, sulfur supply is positively linked to symbiotic nitrogen fixation (SNF) and sulfur starvation causes three additional major effects: decrease of nodulation, inhibition of SNF, and slowing down of nodule metabolism. These effects are due, at least in part, to the impairment of nitrogenase biosynthesis and activity, the accumulation of nitrogen-rich amino acids, and the decline in leghemoglobin, ferredoxin, ATP, and glucose in nodules. During the last decade, some major advances have been made about the uptake and metabolism of sulfur in nodules. These include the identification of the sulfate transporter SST1 in the symbiosomal membrane, the finding that glutathione produced in the bacteroids and host cells is essential for nodule activity, and the demonstration that sulfur assimilation in the whole plant is reprogrammed during symbiosis. However, many crucial questions still remain and some examples follow. In the first place, it is of paramount importance to elucidate the mechanism by which sulfur deficiency limits SNF. It is unknown why homoglutahione replaces glutathione as a major water-soluble antioxidant, redox buffer, and sulfur reservoir, among other relevant functions, only in certain legumes and also in different tissues of the same legume species. Much more work is required to identify oxidative post-translational modifications entailing cysteine and methionine residues and to determine how these modifications affect protein function and metabolism in nodules. Likewise, most interactions of antioxidant metabolites and enzymes bearing redox-active sulfur with transcription factors need to be defined. Solving these questions will pave the way to decipher sulfur-dependent mechanisms that regulate SNF, thereby gaining a deep insight into how nodulated legumes adapt to the fluctuating availability of nutrients in the soil.

## Introduction

In this minireview, we will address the central role of sulfur in symbiotic nitrogen fixation (SNF). The legume-rhizobia symbiosis, the most relevant in agronomical terms, is the result of a complex chemical dialog between the two partners, leading to the formation of unique organs, the nodules, on the roots. During root infection, the bacteria become entrapped within organelle-like structures termed symbiosomes, where the bacteria differentiate into bacteroids. The symbiosomes are surrounded by the symbiosomal membrane, which allows an active metabolic exchange between both symbionts. Bacteroids express the nitrogenase enzyme complex that reduces N_2_ to ammonia. This reaction requires high amounts of ATP and low O_2_ concentrations to prevent the irreversible inactivation of the enzyme. At least two mechanisms maintain a low but steady O_2_ concentration in nodules: an O_2_ diffusion barrier in the mid-inner cortex and leghemoglobin in the cytoplasm of infected cells (Minchin et al., 2008). In functioning nodules, the bacteroids provide the plant cells with ammonium and amino acids, whereas the plant provides the nodule cells with photosynthetically derived sucrose. This sugar is translocated through the phloem from the shoot to the nodules, where it is metabolized to phospho*enol*pyruvate *via* the glycolytic pathway and then to malate by the successive action of phospho*enol*pyruvate carboxylase and malate dehydrogenase. Malate is taken up by the bacteroids and oxidized to CO_2_ and water to obtain the ATP and reducing power required for N_2_ fixation (Naya et al., 2007; Vance, 2008).

The key enzyme of SNF is nitrogenase, which consists of two proteins: an Fe protein (dinitrogenase reductase) and a MoFe protein (dinitrogenase). The Fe protein is a homodimer (γ_2_) encoded by the *nifH* gene and contains a single [4Fe-4S] cluster at the interface of the two subunits, whereas the MoFe-protein is a heterotetramer (α_2_β_2_) encoded by the *nifDK* genes and contains two [8Fe-7S] clusters (P-clusters) at each of the α-β interfaces and one FeMo cofactor [7Fe-9S-Mo-X + *R*-homocitrate] within the active site in each α subunit (Rubio and Ludden, 2008). Thus, nitrogenase is exceptionally rich in sulfur, which suggests that this element may become limiting in symbiosis. Another nodule Cys-rich protein is ferredoxin, an electron transporter that donates electrons to nitrogenase. The protein from *Bradyrhizobium japonicum* bacteroids purified from soybean nodules has two [4Fe-4S] clusters (Carter et al., 1980).

## Sulfur Effects on Legume Plant Growth, Nodulation, and Nitrogen Fixation

**FIGURE 1:**
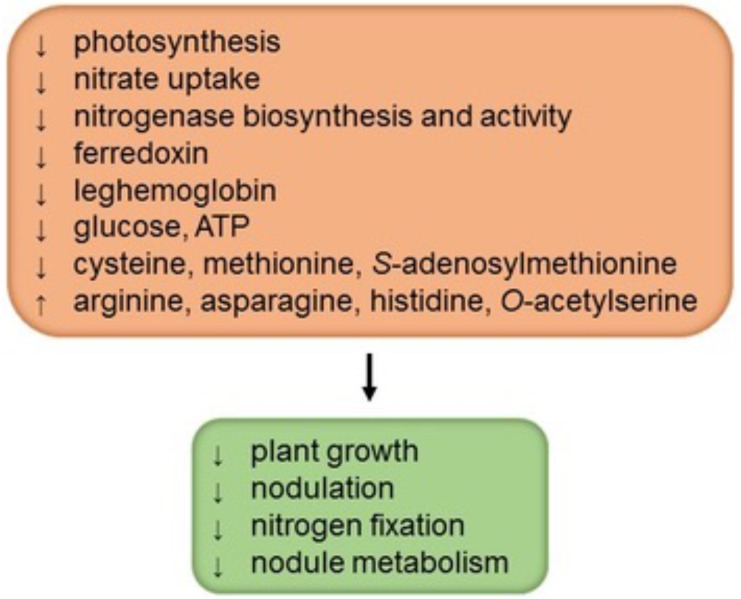
Sulfur deficiency negatively affects the performance of nodulated legumes. The scheme depicts four major general effects in interaction, as well as some specific biochemical features that may, at least in part, account for such effects. Information was gathered from studies by Anderson and Spencer (1950), DeBoer and Duke (1982), Zhao et al. (1999), Scherer (2008), and Varin et al. (2010).

Sulfur is an essential nutrient for plants because it is a constituent of the amino acids cysteine (Cys) and methionine (Met), metal cofactors, coenzymes, and secondary metabolites (reviewed by Davidian and Kopriva, 2010). As occurs in other plants, sulfur deficiency in legumes decreases plant growth, photosynthesis, and yield (**Figure 1**). However, nodulated legumes have a high demand for sulfur and SNF is more sensitive to sulfur deficiency than is nitrate uptake (Zhao et al., 1999; Varin et al., 2010). Not surprisingly then, legumes with a high sulfur supply show greater rates of N_2_ fixation and, conversely, legumes grown on sulfur-poor soils have lower nitrogenase activity and readily respond to sulfur fertilizers by increasing yield and nitrogen content (Anderson and Spencer, 1950; Scherer, 2008). In nodulated legumes sulfur deficiency triggers at least three types of effects: decrease of nodulation, direct inhibition of N_2_ fixation, and general alteration of nodule metabolism (**Figure 1**). In white clover (*Trifolium repens*), the effect of sulfur deficiency on nodulation was evidenced by an important reduction in the nodule number and in the nodule mass per root length; this decrease in nodulation could be attributed to a nitrogen-dependent negative feedback as a result of the high accumulation of nitrogen-rich amino acids (arginine, asparagine, and histidine) in nodulated roots (Varin et al., 2010). Conversely, a high sulfur supply to plants markedly increases nodulation and SNF (Anderson and Spencer, 1950; Varin et al., 2010).

The lower SNF in sulfur-deficient plants may be attributed not only to a decrease in nodulation but also to a direct effect on nitrogenase and a general down-regulation of nodule metabolism. Notably, nodules of sulfur-deficient plants have lower rates of nitrogenase biosynthesis and activity (DeBoer and Duke, 1982; Scherer, 2008; Varin et al., 2010), probably because of a restricted availability of Cys and Met. Also, sulfur-deficient nodules contain less leghemoglobin (in the cytosol), glucose (in whole nodules), ATP (in the mitochondria and bacteroids), and ferredoxin (in the bacteroids) than sulfur-sufficient nodules, which suggests a limitation in the provision of energy and carbon skeletons for SNF (Scherer, 2008; Varin et al., 2010). Unfortunately, the profiles of sugars, amino acids, dicarboxylic acids, and carbon metabolism enzymes in nodules over the course of sulfur deficiency have not been reported. In our opinion, such a study, probably combined with metabolomic and proteomic approaches, will be required to elucidate the mechanism by which sulfur deficiency inhibits SNF.

## Sulfate Uptake and Assimilation in Nodule Host Cells and Bacteroids

**FIGURE 2:**
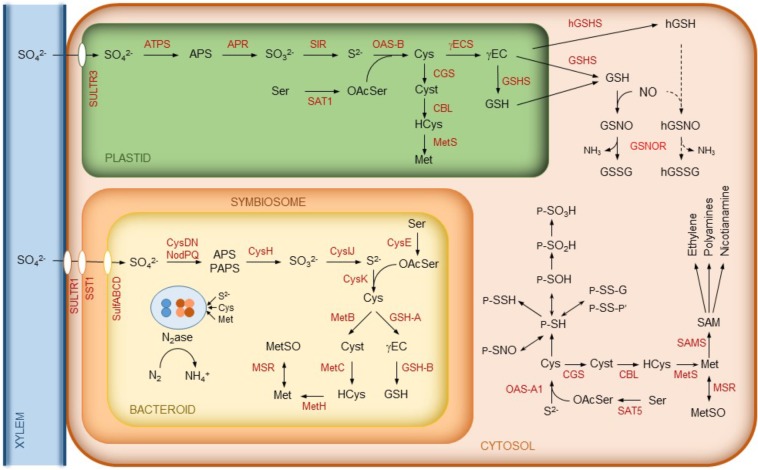
Schematics of sulfur metabolism in legume nodules. In the cytosol, some common reactions affecting the thiol group of Cys residues of proteins (P) are indicated. These include sulfenylation (P-SOH), sulfinylation (P-SO_2_H), sulfonylation (P-SO_3_H), persulfidation (P-S-SH), *S*-nitrosylation (P-SNO), glutathionylation (P-S-S-G, where G is a glutathione molecule linked to a protein Cys residue through its thiol group), and formation of mixed disulfides (P-S-S-P’, where P and P’ may be identical or different proteins). Note that sulfenylation, glutathionylation, and formation of mixed disulfide are reversible, whereas sulfinylation may be irreversible and sulfonylation is irreversible. For simplicity, we omit reactions such as Cys synthesis that may also occur in the mitochondria. Abbreviations are as indicated in **Table 1**. Cyst, cystathionine; (h)GSSG, (homo)glutathione disulfide; HCys, homocysteine; MetSO, methionine sulfoxide; OAcSer, *O*-acetylserine.

Sulfur is taken up as sulfate by plant cells through sulfate transporters and needs to be reduced to organic sulfide (**Figure 2**). The assimilation of sulfate starts with its activation *via* adenylation to adenosine-5′-phosphosulfate (APS) catalyzed by ATP sulfurylase. APS is then successively reduced to sulfite and sulfide by APS reductase (APSR) and sulfite reductase (SIR), respectively. Sulfide is incorporated into *O*-acetylserine by *O*-acetylserine(thiol)lyase (OAS) yielding Cys. In turn, *O*-acetylserine is synthesized from serine and acetyl-coenzyme A by serine acetyltransferase (SAT). Some of these enzymes occur as isoforms localized to different cellular compartments. In *Arabidopsis thaliana*, OAS-A1, OAS-B, and OAS-C are in the cytosol, chloroplasts, and mitochondria, respectively (Heeg et al., 2008). Homolog OAS isoforms have been found in legume nodules (**Figure 2**). Likewise, there are multiple SAT isoforms, including cytosolic SAT5 and plastidic SAT1 (Krueger et al., 2009). Although the SAT1 an SAT5 transcripts are detectable in nodules according to the Gene Expression Atlas of the model legumes *Lotus japonicus* (LjGEA^1^) and *Medicago truncatula* (MtGEA^2^), proteomic analyses failed to detect the enzymes, indicating that they are low abundant.

On the other hand, Met is synthesized from Cys in the plastids by the sequential action of cystathionine γ-synthase (CGS), cystathionine β-lyase (CBL), and methionine synthase (MetS). However, in *A. thaliana* and legumes, there are also cytosolic MetS isoforms that provide Met for the synthesis of ethylene, polyamines, and nicotianamine. This requires the prior activation of Met to *S*-adenosylmethionine by *S*-adenosylmethionine synthase (SAMS) (reviewed by Bürstenbinder and Sauter, 2012). All of the enzymes implicated in the synthesis of Met, ethylene, and polyamines have been detected in nodules and, in some cases, with relatively high abundance (Larrainzar et al., 2007, 2014). For clarity, only some of them are depicted in **Figure 2**. A proteomic quantitative approach revealed that the nodule contents of MetS and SAMS, along with ethylene production, are reduced during drought stress, yet the levels of Cys and Met remain constant. This suggests that the homeostasis of sulfur-containing amino acids is important for SNF (Larrainzar et al., 2007).

Over the last two decades, the role of sulfate transport and metabolism in SNF has attracted increasing attention. The first indication that sulfate is exchanged between the two symbiotic partners came from the proteomic identification of a sulfate transporter in the symbiosomal membrane of *L. japonicus* (Wienkoop and Saalbach, 2003). This transporter was found to be specifically and highly expressed in the symbiosomal membrane, suggesting a major role of active sulfate transport in symbiosis. Krusell et al. (2005) used *L. japonicus* mutants to show that the sulfate transporter is crucial for SNF and they designated it as symbiotic sulfate transporter 1 (SST1). The *sst1* mutants displayed symptoms of N deficiency only under symbiotic conditions. The nodules of the mutant plants exhibited a ∼30% decrease in the contents of leghemoglobin and Fe protein (NifH), as well as a reduction of ∼90% in the rates of N_2_ fixation. Interestingly, Kalloniati et al. (2015) observed that nodules formed by ineffective (Fix^-^) mutant strains of *Mesorhizobium loti* (with no nitrogenase activity) contained considerably lower levels of thiols than the nodules of wild-type (Fix^+^) plants, and the same occurred with the thiol contents of roots and stems. These and other results led these authors to conclude that the nodules are a primary source of sulfur and trigger a complete reprogramming of the whole-plant sulfur metabolism.

Sulfur metabolism is also very active and relatively complex in rhizobia. **Figure 2** shows the key steps of the biosynthesis of Cys and Met in the bacteroids. The first step of Cys synthesis involves the activation of sulfate and may occur through two enzyme complexes, NodPQ and CysDN, yielding APS and 3′-phosphoadenosine-5′-phosphosulfate (PAPS) (Snoeck et al., 2003, and references therein). Then, APS/PAPS is reduced to sulfite by APS/PAPS reductase (CysH). This enzyme has preference for APS over PAPS (Abola et al., 1999). Sulfite is reduced by sulfite reductase (CysIJ) to sulfide, which is incorporated into *O*-acetylserine by cysteine synthase (CysK) to produce Cys. This amino acid may then be used for the synthesis of proteins, Met, and the tripeptide glutathione (GSH; γGlu-Cys-Gly). The synthesis of GSH in the bacteroids proceeds in two steps catalyzed by γ-glutamylcysteine synthetase (GSH-A) and glutathione synthetase (GSH-B), as occurs in plant cells (**Figure 2**). Also, similarly to the pathway in nodule cells, the synthesis of Met from Cys in the bacteroids occurs through three steps catalyzed by cystathionine γ-synthase (MetB), cystathionine β-lyase (MetC), and methionine synthase (MetH) (**Figure 2**; for further details see review by Dunn, 2014).

The information about the transport and/or synthesis of Cys and Met in rhizobia and bacteroids is quite confusing because differences may exist between rhizobial species. A search in the UniProtKB proteome database suggests that rhizobia have no Cys transporters but do have several Met transporters. It can thus be argued that the plant is able to provide Met to the bacteroids, although there is still no evidence for a specific Met transporter in the symbiosomal membrane. Working with bean plants (*Phaseolus vulgaris*), Taté et al. (1999) concluded that Met is produced by *Rhizobium etli* bacteroids and not supplied by the host cells, although it is required for nodule function. In sharp contrast, Abbas et al. (2002) found that mutant Cys auxotrophs of *Sinorhizobium meliloti* form fully effective nodules on alfalfa but Met auxotrophs form ineffective nodules, indicating that the plant host provides Cys, but not Met, to the bacteroids. Thus, although sulfate is probably the major source of sulfur actively transported to the bacteroids *via* the SST1 transporter, it remains to be seen if the plant also provides the bacteroids with sulfur-containing metabolites to support SNF.

A peculiar symbiotic function of sulfur in the bacteroids is the sulfation of nodulation (Nod) factors and of cell surface polysaccharides. Nod factors are lipo-chitooligosaccharide signal molecules that are crucial for the onset of symbiosis because they elicit root hair deformation and nodule organogenesis. Sulfation of Nod factors is catalyzed by the sulfotransferase activity of NodH (Ehrhardt et al., 1995). It is essential for the onset of the alfalfa-*S. meliloti* symbiosis but not of the *L. japonicus*-*M. loti* symbiosis because *M. loti*, unlike *S. meliloti*, does not produce sulfated Nod factor (Cronan and Keating, 2004; Townsend et al., 2006). However, both *S. meliloti* and *M. loti* produce sulfated lipopolysaccharides using PAPS as sulfate donor. In fact, a *M. loti* mutant in which the *nodPQ* gene, required for PAPS biosynthesis, has been inactivated, failed to produce sulfated polysaccharides and showed a reduced ability to elicit nodules in its host legume (Townsend et al., 2006).

## Biosynthesis, Regulation, and Function of Thiols in Nodules

In plants and other organisms, Cys is a precursor of GSH, which is involved in multiple physiological processes of plants such as sulfur transport and storage, cellular redox homeostasis, regulation of the cell cycle, responses to abiotic and biotic stress, and heavy metal detoxification (reviewed by Noctor et al., 2012). In leguminous plants, a structural homolog of GSH, homoglutathione (hGSH; γGlu-Cys-βAla), may partially or completely replace GSH. There is considerable variation in the relative abundance of GSH and hGSH with the legume species and plant organ. Two model legumes, *L. japonicus* and *M. truncatula*, display contrasting patterns of thiol accumulation. *L. japonicus* contains almost exclusively hGSH in roots and leaves but both GSH and hGSH in nodules (Matamoros et al., 2003), whereas *M. truncatula* produces only GSH in leaves and the two thiols in roots and nodules (Frendo et al., 1999). The synthesis of GSH and hGSH takes place by two sequential reactions (**Figure 2**). The first one is catalyzed by γ-glutamylcysteine synthetase (γECS) and the second by glutathione synthetase (GSHS) or homoglutathione synthetase (hGSHS), respectively (Klapheck et al., 1988; Bergmann and Rennenberg, 1993). The hGSHS enzyme exhibits a much higher affinity for β-alanine than for glycine, and this specificity is determined only by two contiguous amino acid residues, leucine and proline, at the active site (Frendo et al., 2001; Iturbe-Ormaetxe et al., 2002; Galant et al., 2011). The *GSHS* and *hGSHS* genes are tandemly arranged on the chromosome in *M. truncatula* (Frendo et al., 2001) and *L. japonicus* (Matamoros et al., 2003). This has led to the proposal that *hGSHS* derives from *GSHS* by gene duplication (Frendo et al., 2001).

In nodules, γECS is localized in the plastids, whereas GSHS and hGSHS are localized in the plastids and cytosol (Moran et al., 2000; Clemente et al., 2012). The concentrations of GSH and hGSH are positively correlated with the respective synthetase activities and are particularly high in the meristems and infected zone (Matamoros et al., 1999). In *M. truncatula* nodules, γECS mRNA is more abundant in the meristems and infection zone, GSHS mRNA in the cortex and fixing zone, and hGSHS mRNA in the cortex (El Msehli et al., 2011). The two genes are also differentially expressed in response to stress, hormones, nitric oxide (NO), and other signaling compounds (Innocenti et al., 2007; Clemente et al., 2012). This strongly suggests that GSH and hGSH may have at least some distinct functions. This issue will be only set, however, with the help of mutants deficient specifically in GSHS or hGSHS.

Several lines of evidence underline the importance of GSH for SNF. The nodule concentration of GSH (or hGSH) and N_2_-fixing activity are positively correlated and both decline with nodule senescence (reviewed by Puppo et al., 2005; Becana et al., 2010; Frendo et al., 2013). Moreover, nodules contain higher concentrations of free Cys and GSH (or hGSH) than roots and leaves (Frendo et al., 1999; Matamoros et al., 1999; Kalloniati et al., 2015). Roots depleted of GSH because its synthesis has been blocked with buthionine sulfoximine or because they express *GSHS* in antisense orientation show few nascent nodules and lower rates of N_2_ fixation; conversely, the overexpression of γECS increased GSH content and N_2_ fixation (Frendo et al., 2005; El Msehli et al., 2011). Bacteroids contain most of the GSH (∼85%) of nodules (Matamoros et al., 2013), consistent with the high expression of the genes of the GSH biosynthetic pathway (Roux et al., 2014). Mutants (*gshA*) of *S. meliloti* lacking γECS did not nodulate *M. truncatula* (Harrison et al., 2005), whereas the equivalent mutants of *Bradyrhizobium* sp. were able to form effective (Fix^+^) nodules in peanut (Taté et al., 2012), which suggests that in the latter case the plant may compensate the bacteroids for the lack of GSH. As for the second enzyme of the pathway, the *S. meliloti* mutants (*gshB*) formed nodules having 75% less N_2_ fixation (Harrison et al., 2005) and those of *Rhizobium tropici* formed nodules that displayed early senescence (Muglia et al., 2008). Collectively, these observations support a central role of GSH, produced by the bacteroids and the plant, in legume nodules.

The tripeptides GSH and hGSH play a role in metal detoxification as precursors of phytochelatins and homophytochelatins (Loscos et al., 2006). These polypeptides are rich in Cys and rapidly accumulate in plant organs, especially in the roots, upon treatment with cadmium and other heavy metals and metalloids. They chelate metal ions through the Cys residues and the complexes are transported into the vacuoles, avoiding cellular toxicity. The accumulation of (homo)phytochelatins is caused by metal-triggered activation of (homo)phytochelatin synthases, which are expressed in most legume tissues including the nodules (Loscos et al., 2006).

In addition to thiol metabolites, some enzymes have catalytic thiol groups that confer them important roles as antioxidants and signal transmitters. Thiol peroxidases are non-heme proteins encoded by large multigene families that include peroxiredoxins (Prxs) and glutathione peroxidases (Gpxs) (reviewed by Rouhier et al., 2008). There are four classes of Prxs (1-Cys, 2-Cys, type II, and type Q) that catalyze the reduction of H_2_O_2_ or hydroperoxides to water or the corresponding alcohols, respectively. On the other hand, plant Gpxs usually act as phospholipid hydroperoxidases reducing lipid peroxides. Both Prxs and Gpxs use thioredoxins (Trxs) as major reductants. In *L. japonicus*, we identified seven *Prx* and six *Gpx* genes (Ramos et al., 2009; Tovar-Méndez et al., 2011). In nodules, we found significant amounts of cytosolic PrxIIB and mitochondrial PrxIIF (Tovar-Méndez et al., 2011), as well as of Gpx1 in the plastids and nuclei and Gpx3 in the cytosol and endoplasmic reticulum (Matamoros et al., 2015).

Other enzymes of utmost importance in Cys-mediated antioxidant protection and redox regulation are Trxs, glutaredoxins (Grxs), and glutathione transferases (GSTs). Many isoforms of these enzymes have been detected in nodules (**Table 1**). In *L. japonicus*, we identified 14 Trxs and three NADPH-thioredoxin reductases (NTRs). Most of them are expressed in nodules, which suggests the functioning of an NTR-Trx-Prx-Gpx redox system in nodules (Tovar-Méndez et al., 2011). In *M. truncatula*, two novel Trx isoforms (Trx*s1* and Trx*s2*) have been recently identified (Ribeiro et al., 2017). They are mainly expressed in the nodules and Trx*s1* is targeted to the symbiosomes, where it interacts with nodule-specific Cys-rich peptides (NRCs) that control terminal rhizobial differentiation. Interestingly, these peptides have potential antimicrobial properties but do not kill the bacteroids (Maróti et al., 2015).

**Table 1:** Proteins involved in Cys and Met metabolism in the host cells and bacteroids of legume nodules.

Enzyme	Abb^a^	Examples	Reference^b^
**Plant Cys metabolism**			
Sulfate transporter	SULTR	SULTR1, SULTR3	(mRNA)
Symbiotic sulfate transporter 1	SST1	SST1	Wienkoop and Saalbach, 2003
ATP sulfurylase	ATPS	ATPS	Larrainzar et al., 2007
Adenosine 5′-phosphosulfate reductase	APR	APR	Kalloniati et al., 2015
Sulfite reductase	SIR	SIR	(mRNA)
*O*-Acetylserine(thiol)lyase	OAS	OAS-A1, OAS-B	Larrainzar et al., 2007; Dam et al., 2014
Serine acetyltransferase	SAT	SAT2, SAT3	(mRNA)
γ-Glutamylcysteine synthetase	γECS	γECS	Frendo et al., 1999; Matamoros et al., 1999
Glutathione synthetase	GSHS	GSHS	Frendo et al., 1999; Matamoros et al., 1999
Homoglutathione synthetase	hGSHS	hGSHS	Frendo et al., 1999; Matamoros et al., 1999
Peroxiredoxins	Prxs	PrxIIB, PrxIIF	Larrainzar et al., 2007; Tovar-Méndez et al., 2011; Dam et al., 2014
Glutathione peroxidases	Gpxs	Gpx1, Gpx3	Matamoros et al., 2015
Thioredoxins	Trxs	Trx*h1*, Trx*o*, Trx*s1*, Trx*s2*	Larrainzar et al., 2007; Dam et al., 2014; Ribeiro et al., 2017
NADPH-thioredoxin reductases	NTRs	NTRA, NTRB, NTRC	Tovar-Méndez et al., 2011; Dam et al., 2014
Glutaredoxins	Grxs	GrxC2, GrxC4	Larrainzar et al., 2007; Tovar-Méndez et al., 2011; Marx et al., 2016
Glutathione transferases	GSTs	GST9, GST15, GST22	Larrainzar et al., 2007; Dalton et al., 2009; Dam et al., 2014
Cysteine-rich peptides	NRC	NRC247, NRC335	Maróti et al., 2015; Ribeiro et al., 2017
**Plant Met metabolism**			
Cystathionine γ-synthase	CGS	CGS	Larrainzar et al., 2014
Cystathionine β-lyase	CBL	CBL	Larrainzar et al., 2014
Methionine synthase	MetS	MetS1, MetS2, MetS3	Larrainzar et al., 2007, 2014
*S*-Adenosylmethionine synthase	SAMS	SAMS	Larrainzar et al., 2007
**Bacteroid Cys metabolism**			
Sulfate binding transporter (ABC transporter)	SulABCD	SulABCD	Cheng et al., 2016
ATP sulfurylase	NodPQ/CysDN	NodPQ/CysDN	Abola et al., 1999
Serine acetyltransferase	CysE	CysE	Parker et al., 2001
Adenosine 5′-phosphosulfate reductase	CysH	CysH	Abola et al., 1999; Bick et al., 2000
Sulfite reductase	CysJ	CysJ	(mRNA)
Cysteine synthase	CysK	CysK	Dunn, 2014
γ-Glutamylcysteine synthetase	GSH-A	GSH-A	Harrison et al., 2005
Glutathione synthetase	GSH-B	GSH-B	Harrison et al., 2005; Larrainzar et al., 2007; Muglia et al., 2008; Taté et al., 2012
Glutaredoxins	Grxs	Grx1, Grx2	Benyamina et al., 2013
**Bacteroid Met metabolism**			
Cystathionine γ-synthase	MetB	MetB	Dunn, 2014
Cystathionine β-lyase	MetC	MetC	Dunn, 2014
Methionine synthase	MetH	MetH	Dunn, 2014

*^*a*^Some common abbreviations.^*b*^References of studies where the protein and/or enzyme activity has been detected. To our knowledge, for some proteins, marked as “(mRNA)”, there are only available studies at the mRNA level*.

The Grx enzyme family is still more complex, with ∼30 isoforms identified in vascular plants (Rouhier et al., 2008). Grxs are GSH-dependent enzymes that act as redox regulators by directly reducing peroxides or dehydroascorbate; they play also a role in sulfur metabolism through their participation in (de)glutathionylation reactions (Rouhier et al., 2008) and in the assembly of [Fe-S] clusters (Moseler et al., 2015). Proteomic analyses allowed us to identify two Grxs (GrxC2 and GrxC4) in *L. japonicus* nodules (Tovar-Méndez et al., 2011). Regarding the bacterial partner, the *S. meliloti* genome encodes three Grxs; two of them, SmGrx1 and SmGrx2, are essential for bacteroid differentiation, nodule development, and N_2_ fixation capacity (Benyamina et al., 2013).

Finally, GSTs constitute a ubiquitous superfamily of enzymes, with 25 genes described in soybean (*Glycine max*) and 47 in *A. thaliana*. They catalyze the conjugation of xenobiotics and secondary metabolites with GSH (and probably of hGSH); the GSH-conjugates are then imported into the vacuoles by ATP-binding cassette transporters and degraded (reviewed by Dixon et al., 2010). In soybean nodules, there are 14 GST isoforms, of which GST9 is predominant. The levels of this isoform are enhanced in senescent nodules and its down-regulation results in a decrease of SNF and an increase in carbonylated proteins, which indicates that GSTs may, at least in part, act as antioxidants protecting nodule activity (Dalton et al., 2009). Reinforcing this view, we noticed a startingly high number of GSTs in the rhizobial proteomes (UniProtKB).

## Post-Translational Sulfur-Related Modifications and Redox Signaling in Nodules

The Cys side chain is a potent nucleophile that readily reacts with oxidants and electrophilic species. In proteins, the thiol group often plays an important role in catalysis and is a major site of post-translational modifications (PTMs) that include oxidation to disulfide (-S-S-), sulfenic (-SOH), sulfinic (-SO_2_H), and sulfonic (-SO_3_H) acids; *S*-nitrosylation (-SNO); persulfidation (-SSH); and glutathionylation (-SS-glutathione) (**Figure 2**). These properties make the thiol group a major actor in intracellular redox signaling (Rouhier et al., 2008; Kovacs and Lindermayr, 2013; Aroca et al., 2015; Waszczak et al., 2015).

A study based on the use of chemical and genetic probes that specifically trap sulfenic acid on Cys residues allowed the identification of sulfenylated proteins at different stages of the *M. truncatula*–*S. meliloti* symbiosis (Oger et al., 2012). In roots 2 days after infection, sulfenylated proteins were found to be mostly related to redox processes, whereas in mature nodules the sulfenylated proteins detected were mainly involved in amino acid and carbohydrate metabolism. In addition, bacteroid proteins involved in N_2_ fixation were also identified as sulfenylated. These results suggest that the establishment of symbiosis and nodule metabolism are regulated by selective protein oxidation (Oger et al., 2012).

The information on protein *S*-nitrosylation in nodules is still scant. Nodules contain GSH and hGSH that react with NO to yield *S*-nitrosogluthathione and *S*-nitrosohomoglutathione (**Figure 2**). These *S*-nitrosothiols may act as NO reservoirs and donors in nitrosylation reactions. The enzyme *S*-nitrosoglutathione reductase (GSNOR) catalyzes the breakdown of *S*-nitrosogluthathione and thereby modulates protein *S*-nitrosylation (Xu et al., 2013). The presence of GSNOR transcripts in nodules [LjGEA gene ID: Lj1g3v4528570] and of enzyme activity in *S. meliloti* and *Bradyrhizobium* sp. (Maiti et al., 2012) suggests a role for *S*-nitrosylation in nodules. Proteomic analyses identified 80 nitrosylated proteins in mature nodules of *M. truncatula* (Puppo et al., 2013). Of these, 27 proteins, mostly involved in carbohydrate metabolism and in the tricarboxylic acid cycle, were also found as sulfenylated. Notably, SAMS, which is an abundant enzyme of nodules and a crucial link of Met with ethylene and polyamine synthesis (Larrainzar et al., 2014), is inhibited *in vitro* by nitrosylation (Lindermayr et al., 2006). The inhibitory effect was specific for the SAMS1 isoform, and it will be thus very important to ascertain whether nitrosylation of SAMS1 plays a distinct regulatory role *in vivo* compared with the other two isoforms (SAMS2 and SAMS3). Many other enzymes directly implicated in sulfur metabolism (APS, SIR, MetS, and OAS) or related to it (γECS and several isoforms of Gpx, Grx, and GST) were found also to be nitrosylated in mutant plants (*gsnor1-3*) defective in GSNOR (Hu et al., 2015), clearly evidencing a central role of GSNOR in nitrosothiol homeostasis. Furthermore, in nodules of *L. japonicus*, Gpx1 and Gpx3 were found to be nitrosylated. The modification of the peroxidatic Cys resulted in a partial inhibition of the enzyme activity, suggesting that these proteins participate in the crosstalk between reactive oxygen species and NO (Matamoros et al., 2015). All these observations strongly suggest that *S*-nitrosylation is a major PTM controlling sulfur metabolism and associated antioxidant and redox-regulating enzyme activities. The presence of glutathionylated (Rouhier et al., 2008) or persulfidated (Aroca et al., 2015) proteins in nodules has not been demonstrated so far. However, it seems likely that these PTMs will also regulate the functions of certain proteins of nodules in a similar manner to that described in animal cells and in other plant organs (Aroca et al., 2015, and references therein).

Together with Cys, sulfur-containing Met is the amino acid most susceptible to oxidation. The process is termed Met sulfoxidation and yields a mixture of Met-S-sulfoxide and Met-R-sulfoxide. The reduction back to Met is catalyzed by two methionine sulfoxide reductases, MsrA and MsrB, that reduce, respectively, the S and R epimers. These enzymes are present in most organisms, from bacteria to humans, and are emerging as novel regulators of protein function in plant cells (Tarrago et al., 2009). The ratios of Met to Met sulfoxide remain stable in most proteins from bean nodules during aging (Matamoros et al., 2013). One exception is glutamine synthase, a key enzyme of nodule carbon and nitrogen metabolism. It was found that the isoform GS-N1 contains two Met residues that are oxidized to sulfoxides in senescent nodules. The precise effect of this modification on protein function, however, could not be determined. A search in the rhizobial proteome databases also retrieved several peptide-methionine sulfoxide reductases, such as MsrA and MsrB. They regulate the reversible sulfoxidation of Met using Trx, but, to our knowledge, these enzymes have not been fully characterized.

## Conclusion

Compelling evidence has accumulated to conclude that sulfur metabolism is of paramount importance for SNF. Some breakthroughs have been the identification of the symbiosomal SST1 transporter, the finding that GSH produced by both symbiotic partners is critical for nodule functioning, and the demonstration that sulfur assimilation in plant tissues is reprogrammed during the onset of symbiosis. Yet it is imperative to define the mechanism by which sulfur deficiency limits SNF, establishing the time course of molecular and cellular events, and to ascertain whether such a mechanism is conserved between legumes having indeterminate and determinate nodulation. To this end, it seems critical to determine the complete profiles of metabolites and the activities of carbon metabolism enzymes in nodules, as well as to elucidate how the transport and assimilation of sulfate is regulated inside the nodules. Likewise, it is important to ascertain the differences in sulfur metabolism between indeterminate and determinate nodules. An outstanding example is the presence, exclusively in indeterminate nodules, of symbiotic-specific Trxs that regulate the redox state of Cys-rich peptides involved in bacteroid differentiation. Information is also lacking about the synthesis and degradation of Cys and GSH in the bacteroids and nodule host cells. To date there is no explanation why hGSH replaces GSH only in some legumes and tissues within the same legume species. There are very few studies aimed at identifying oxidative PTMs of Cys and Met residues and their impact on protein function in nodules. Two of them, glutathionylation and persulfidation, have not reported so far in bacteroid and/or host cell proteins of nodules. Also, most of the interactions of (h)GSH, Prxs, Gpxs, and Trxs with enzymes and transcription factors are yet to be defined. Addressing all these questions will result in the discovery of novel regulatory mechanisms involved in SNF and in the adaptation of nodulated legumes to changing environmental conditions.

## Author Contributions

MB and MM wrote the manuscript. SW performed proteome database mining and provided helpful information. All authors agreed with submission.

## Conflict of Interest Statement

The authors declare that the research was conducted in the absence of any commercial or financial relationships that could be construed as a potential conflict of interest.

## Footnotes

1 https://lotus.au.dk/expat/

2 https://mtgea.noble.org/v3/
